# Do language families matter? Evaluating LLMs for sentiment analysis through a hierarchical cross-lingual lens

**DOI:** 10.3389/frai.2026.1854873

**Published:** 2026-07-24

**Authors:** Muhamet Kastrati, Abdul Manaf, Ali Shariq Imran, Zenun Kastrati, Sher Muhammad Daudpota, Marenglen Biba

**Affiliations:** 1 Department of Computer Science, University of New York Tirana, Tiranë, Albania; 2 Department of Computer Science, Sukkur IBA University, Sukkur, Pakistan; 3 Department of Computer Science, Norwegian University of Science and Technology, Gjøvik, Norway; 4 Department of Informatics, Linnaeus University, Växjö, Sweden

**Keywords:** few-shot, language family, large language models, low-resource languages, sentiment analysis, social media, zero-shot

## Abstract

Social media sentiment analysis has become one of the most significant instruments for understanding the opinion of the population in the spheres of healthcare, politics, and education. Yet, large language models (LLMs) remain unevenly distributed in their linguistic coverage, failing to adequately serve a large portion of the world's languages. This study evaluates five state-of-the-art LLMs: GPT-4o, Gemini 2.0 Flash, DeepSeek-V3, Mistral Large, and Claude 3.7 Sonnet on three-class sentiment classification across 36 datasets spanning 36 languages, with emphasis on low- and medium-resource settings, using zero-shot and few-shot prompting without task-specific fine-tuning. In addition to the traditional measures of performance per language, the study presents a hierarchical analysis of languages based on a genealogical tree of Indo-European, Afro-Asiatic, Niger-Congo, Turkic, Austronesian, and English Creole language families, so that it is possible to identify the systematic patterns of performance superiority and inferiority among the language families. The findings show that few-shot prompting improves the results of a vast majority of languages, with several models approaching or surpassing the performance of the state-of-the-art benchmark of task-specific models. The GPT-4o and Claude achieved the highest performance in the high-resource and medium-resource settings, and Gemini is a competent trade-off that allows balancing the performance and the computational cost. Although it has lower zero-shot performance, Mistral benefits the most from few-shot prompting and becomes highly competitive in the few-shot setting. Despite these developments, the level of performance on low-resource languages, such as Oromo, Xitsonga, Azerbaijani, and Twi, remains significantly lower, underscoring that progress in multilingual LLMs requires moving beyond English-centric evaluation toward genuinely representative and globally inclusive benchmarks.

## 1 Introduction

LLMs have recently gained tremendous popularity from both industry and academic audiences due to their effectiveness and strong performance across a wide range of applications (Chang et al., 2024). These models are becoming the preferred approach for a range of natural language processing (NLP) tasks, including machine translation, text summarization, sentiment analysis and question-answering, just to name a few. In particular, thanks to their capability to capture nuanced linguistic patterns, LLMs such as ChatGPT show great potential to address sentiment analysis problems (Zhang et al., 2023). However, the development of LLMs has primarily focused on high-resource languages, particularly English, while performance on medium- and especially low-resource languages remains poor. This creates a considerable gap in supporting the many low-resource languages, as they constitute the majority of the world's languages (Lupascu et al., 2025).

Sentiment analysis is a research field in NLP that extracts users' opinions from target text and determines whether the sentiment is positive, negative, or neutral (Kastrati et al., 2023). The study of sentiment and emotion analysis in text within the context of online social networks, especially Twitter and Facebook, has been of growing interest to researchers in recent years. The impressive amount of textual content created by the users of social media is a precious source of defining personal feelings and emotions. The sentiment analysis of small textual utterances, including those that are presented on the social platform, has a profound impact on a wide range of industries, particularly healthcare, education, and the governmental sector in general (Kastrati et al., 2024).

According to Kastrati et al. (2022), over the last two decades, a variety of learning paradigms have been used to perform sentiment analysis, including lexicon-based and rule-based methods, standard machine learning algorithms, and SOTA deep-learning networks. ChatGPT has brought large language models (LLMs) to the forefront, and numerous studies have focused on LLMs, specifically in their ability to perform a range of NLP tasks, including sentiment classification. As a result, conventional NLP techniques are increasingly being replaced by LLMs in various text-based applications, including the classification of sentiment and emotions, among others (Kastrati et al., 2025). For LLMs to be globally inclusive and relevant, they must perform reliably across all linguistic and cultural contexts, not just in dominant languages like English, French, Mandarin, and Spanish. On the other hand, SOTA models are more biased toward high-resource languages, which is due to the large corpus of available data, annotated datasets, and computation. Whereas medium- and low-resource languages have less availability of corpora data, which leads to limited LLM performance in those configurations (Lupascu et al., 2025).

To address this gap, we present a large-scale assessment of five of the most prominent LLMs, including GPT-4o, Gemini, DeepSeek, Mistral, and Claude, for sentiment analysis on social media platforms, with a particular focus on low-resource languages. The analysis includes 36 datasets, which are 36 different languages, and they represent a wide range of linguistic and cultural backgrounds. The findings have shown that, despite the fact that the considered models have been shown to perform well on both high- and mid-resource languages, their effectiveness varies considerably, with poor performance on languages with scarce training data compared to high-resource languages.

One of the main weaknesses of traditional multilingual benchmark studies, however, is the fact that the reporting of performance metrics is done in a per-language basis, thus individualizing the language. Although there are informative results in such a methodology, it does not reveal the interlinguistic structural dependencies and thus cannot reflect systematic patterns of advantage or disadvantage associated with showing up at the level of language families. This study addresses that gap by combining conventional per-language evaluation with a hierarchical, language-family perspective. We evaluate five widely used LLMs on tasks spanning low-, medium-, and high-resource languages, then organize and analyze results according to a hierarchical language-family tree. Specifically, we organize all 36 languages along a genealogical language-family tree, grouping them by shared linguistic ancestry into families such as Indo-European, Afro-Asiatic, Niger-Congo, Turkic, and Austronesian, and analyze LLM performance at each level of this hierarchy. In this methodological approach, it becomes possible to investigate the possibility of performance clustering based on genealogical affiliation and to identify families that are systematically advantaged or disadvantaged by the existing multilingual training regimes.

This paper also highlights the necessity of putting more focus on the underrepresented languages in research and calls upon the large language model developers to develop inclusive and equitable natural language processing systems that can act in accordance with the global sustainability objectives, namely Sustainable Development Goal 10 (Reduced Inequalities) as defined by the United Nations (2015).

### 1.1 Contributions

The main contributions of this study include:

- Evaluation of five state-of-the-art large language models (GPT-4, Gemini, DeepSeek, Mistral, and Claude) across 36 datasets/languages, including high-, medium-, and low-resource languages across diverse linguistic and cultural contexts.
- Assessment of LLMs under zero-shot and few-shot settings to measure generalization and robustness across diverse languages.
- Introduction of a novel analytical lens-hierarchical language-family aggregation that groups languages by genealogical subtrees to assess patterns of cross-lingual transfer and error concentration.

## 2 Related work

### 2.1 LLMs for sentiment analysis in high-resource languages

The ecosystem of sentiment analysis has an advantage due to recent advancements in the field of LLMs, and it has a strong influence in high-resource languages with richly annotated corpora and robust NLP toolchains. In the early days, sentiment analysis was done primarily through lexicon-based and rule-driven models. Afterwards, supervised machine learning was implemented, and finally, deep learning systems came into the picture. The development of transformer-based models, including BERT and its variants like RoBERTa and DeBERTa, is a critical breakthrough in the performance of classifications. This leads to domain-adaptive fine-tuning that achieves a performance close to human levels in such tasks as financial and product-review sentiment analysis (Wankhade et al., 2022).

In recent years, the development of generative AI, especially LLMs like GPT-3.5 or GPT-4, has placed them as the main tool in sentence classification in English and other high-resource languages due to their sophisticated understanding of the context and ability to engage in zero-shot learning. Zhang et al. (2023) give a detailed assessment of the performance of LLM on sentiment tasks and indicate that, despite great generalization capabilities of models like ChatGPT, models remain inferior to domain-specific fine-tuned models in various assessment scenarios. The increasing use of LLMs across a range of non-English high-resource language settings has drawn scholarly attention. The empirical literature has examined GPT-4 and Claude-3 for vaccine-related sentiment on platforms like Twitter, Reddit, and YouTube in English and found that GPT-4 consistently outperforms smaller models in zero-, one-, and few-shot settings (Annan et al., 2025). Shaikh et al. (2023) used ChatGPT to analyze student feedback sentiment in the educational field. At the same time, Lossio-Ventura et al. (2024) applied ChatGPT to the data of COVID-19 survey questions and compared it to using fine-tuned open pre-trained transformers. Fu et al. (2024) tested GPT-3.5 and GPT-4 specifically in Cantonese, a relatively more resourceful variety, and showed that LLMs could be useful in sentiment problems in non-English settings. All these works confirm the usefulness of LLMs in sentiment classification in high-resource conditions, but the timely design and model selection remain essential factors of performance.

### 2.2 Multilingual and cross-lingual sentiment analysis with LLMs

Multilingual and cross-lingual sentiment analysis aims to transfer sentiment knowledge from high-resource languages (mainly English) to other languages by using cross-linguistic transfer and similar representations (Chen et al., 2015; Jefry et al., 2025; Pelicon et al., 2020). Some studies Balahur and Turchi (2012) and Conneau et al. (2018) used machine translation to translate non-English texts into English before classification. In contrast, modern methods have been based on multilingual pretrained models, including mBERT, XLM-R, and mT5, which are trained on corpora of dozens of languages. These models have shown high zero-shot cross-lingual transfer, enabling direct inference on languages not observed during fine-tuning (Van Thin et al., 2023). However, the degradation in performance has been observed to be significant when using morphologically complex languages or typologically distant languages, and there is always a gap between model performance in English and other languages.

Recently, there has been a surge of research about the use of LLMs to multilingual and cross-linguistic sentiment analysis. Miah et al. (2024) suggested an ensemble that combines transformers and an LLM, where English translation is used as an intermediate step when classifying sentiments in Arabic, Chinese, French, and Italian. Zhu et al. (2024) compared various LLMs on cross-lingual sentiment analysis, pointing to their abilities of cross-lingual knowledge transfer without language-specific training. Within the context of aspect-based sentiment analysis (ABSA), Wu et al. (2025) have performed an extensive study of zero-shot multilingual ABSA with LLMs in English, French, Spanish, Dutch, and Russian, revealing that although the latter have potential, they tend to underperform fine-tuned, task-related models and that simpler zero-shot prompts tend to perform well, especially in high-resource languages. One of the main observations made in these studies is that few-shot prompting can reduce the performance gap significantly between language pairs, although there is still an uneven cross-linguistic performance, with morphologically different languages being the most challenging (Chen et al., 2025).

### 2.3 Sentiment analysis in low-resource and underrepresented languages

Although LLMs are relatively new, a considerable amount of literature has been published on their application to sentiment analysis. For instance, Shaikh et al. (2023) investigated the capabilities of ChatGPT for analyzing student feedback. Similarly, other studies Belal et al. (2023) and Lossio-Ventura et al. (2024) and Fu et al. (2024) have explored the potential of ChatGPT in sentiment analysis. Belal et al. (2023) investigated the use of ChatGPT for data labeling across various sentiment analysis tasks. Lossio-Ventura et al. (2024) compared zero-shot sentiment classification using ChatGPT with few-shot fine-tuning of Open Pre-trained Transformer (OPT), evaluating their performance against traditional sentiment analysis tools on COVID-19 survey data. Further, Fu et al. (2024) evaluated the performance of GPT-3.5 and GPT-4 for analysis of sentiment in Cantonese texts.

Despite the growing body of work, research on sentiment analysis using LLMs for non-English and low-resource languages remains limited. There are a few studies that have specifically addressed sentiment analysis on low-resource languages. For instance, Kastrati et al. (2025) investigated the performance of five prominent LLMs (GPT-3.5 Turbo, GPT-4 Turbo, PaLM 2, and Gemini Pro) on six basic NLP tasks, including analysis of sentiment, using various datasets comprising 12 languages spanning low-, medium-, and high-resource categories. Miah et al. (2024) proposed an ensemble model combining transformers and an LLM, leveraging translation into English to conduct sentiment analysis on different languages such as Arabic, Chinese, French, and Italian. Meanwhile, Sibitenda et al. (2024) integrated rule-based tools with LLMs, particularly LLaMA, to analyze sentiment in African social media posts.

AfriSenti-SemEval shared task (Muhammad et al., 2023) was a pioneering effort in this field, providing annotated Twitter data in 14 African languages and generating a wide range of system submissions. Involved systems made use of multilingual LLMs, including AfroXLM-R and mDeBERTa; the best systems indicated that Africa-centric pretraining and adaptive fine-tuning provide substantial performance improvements on the low-resource settings (García-Díaz et al., 2023). Aliyu et al. (2024) compared the performance of different transformer architectures, such as AfriBERTa, across 12 African languages in the AfriSenti corpus. Their findings showed that AfriBERTa achieved an F1 score of approximately 81%. Recently, Koto et al. (2024) suggested a multilingual sentiment lexicon pretraining strategy that involves 34 languages, determining that zero-shot sentiment classification in low-resource languages can be significantly enhanced without sentence-level target language annotations. Similarly, comparative evaluation of open-source and closed-source LLMs on Bengali, demonstrated that GPT-4o, with few-shot chain-of-thought prompts, had the best F1 score in a variety of NLP tasks. However, open-source models were significantly worse (Nazi et al., 2025). Together, these results highlight the potential and the ongoing constraints of modern LLMs on the underrepresented languages, thus stimulating the general cross-lingual assessment of the present research.

## 3 Methodology

**Figure 1:**
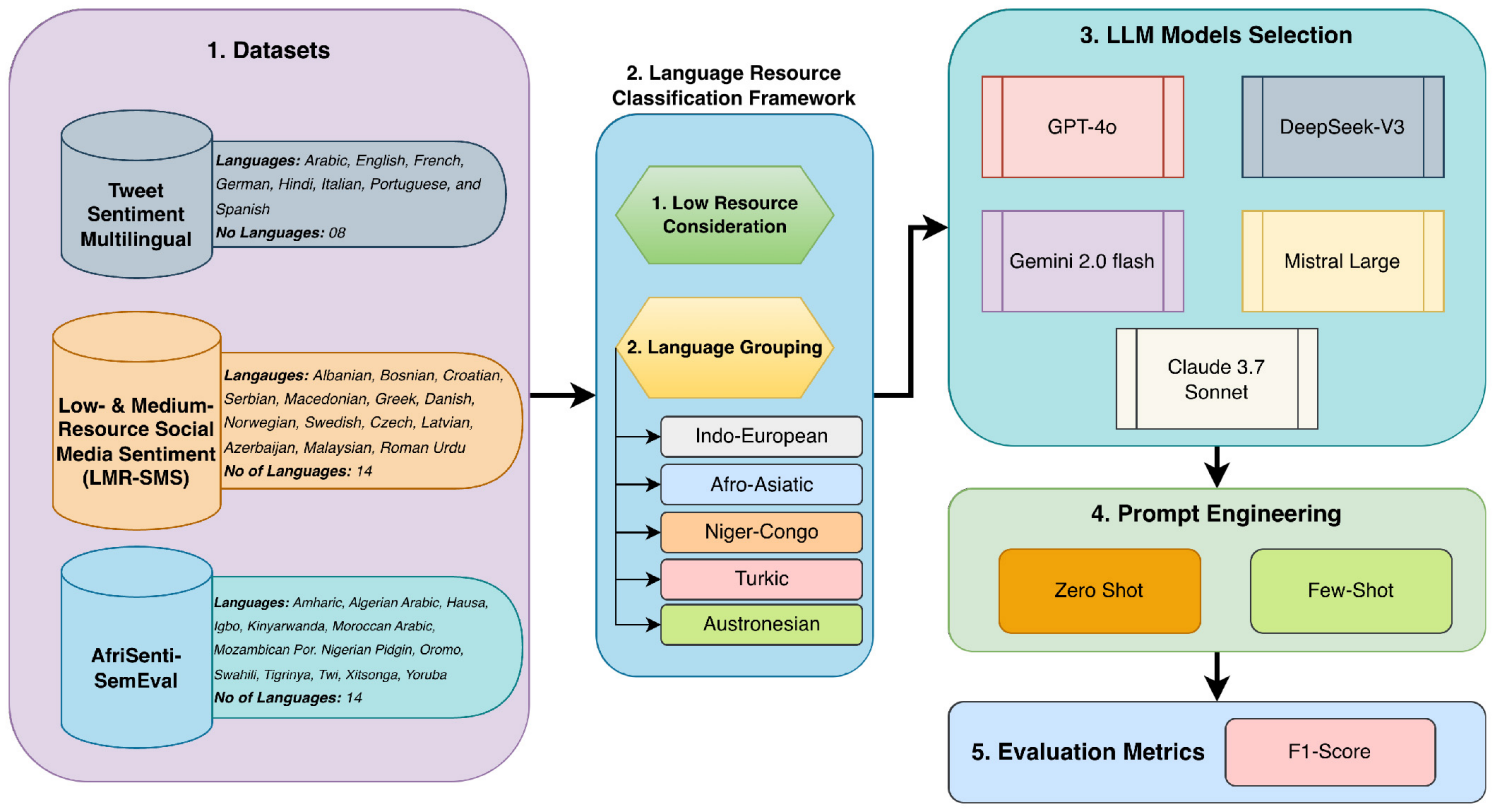
Proposed multilingual sentiment analysis framework showing datasets, language grouping, LLM selection, prompting strategies, and F1-score evaluation.

In this section, we describe the methodology employed in this study. It comprises five main steps, as illustrated in Figure 1, including dataset collection, language resource classification, LLM selection, prompt engineering, and evaluation metrics. Together, these steps form a systematic, large-scale evaluation framework spanning 36 languages, five frontier LLMs, two prompting paradigms, and a novel hierarchical language-family analytical lens. This design provides a reproducible and deployment-realistic benchmark that addresses a significant gap in multilingual NLP evaluation.

### 3.1 Datasets

This section provides a brief overview of the three multilingual sentiment datasets used in this study, covering high-, medium-, and low-resource languages.

#### 3.1.1 Tweet sentiment multilingual

Tweet Sentiment Multilingual (TSM) (Barbieri et al., 2022) is a sentiment-labeled Twitter dataset covering eight languages: Arabic, English, French, German, Hindi, Italian, Portuguese, and Spanish, (sourced from Hugging Face^1^). For our experiments, we used test sets of 870 tweets for each language, annotated with positive, negative, or neutral sentiment.

#### 3.1.2 Low- and medium-resource social media sentiment (LMR-SMS)

LMR-SMS comprises 14 datasets that belong to the low- and medium-resource languages spanning diverse linguistic families and regions.

All datasets consist of publicly available tweet data, except for two (Albanian and Czech) that contain Facebook posts, and are annotated for sentiment in their respective languages. LMR-SMS aims to support multilingual and cross-lingual sentiment analysis research in low-resource settings, offering a benchmark for model evaluation and transfer learning.

In our experiments, the number of instances corresponds either to the test set available on Hugging Face or, in certain cases, to 20% of the entire dataset. Further details regarding the number of instances used for each dataset and language are provided in Table 1.

**Table 1:** Overview of the datasets and their principal characteristics.

S.no.	Language	Source	References	ISO code	Cls.T.	Size	Used	Selection
1	Arabic	TSM	(Barbieri et al., 2022)	ara	3C	3,030	870	HF-t
2	English	TSM	(Barbieri et al., 2022)	eng	3C	3,030	870	HF-t
3	French	TSM	(Barbieri et al., 2022)	fra	3C	3,030	870	HF-t
4	German	TSM	(Barbieri et al., 2022)	deu	3C	3,030	870	HF-t
5	Hindi	TSM	(Barbieri et al., 2022)	hin	3C	3,030	870	HF-t
6	Italian	TSM	(Barbieri et al., 2022)	ita	3C	3,030	870	HF-t
7	Portuguese	TSM	(Barbieri et al., 2022)	por	3C	3,030	870	HF-t
8	Spanish	TSM	(Barbieri et al., 2022)	spa	3C	3,030	870	HF-t
9	Albanian	LMR-SMS	(Kastrati et al., 2021)	alb	3C	10,742	2,148	Test 20%
10	Bosnian	LMR-SMS	(Mozetič et al., 2016)	bos	3C	3,909	782	Test 20%
11	Croatian	LMR-SMS	(Mozetič et al., 2016)	hrv	3C	13,952	2,790	Test 20%
12	Serbian	LMR-SMS	(Mozetič et al., 2016)	srb	3C	11,358	2,272	Test 20%
13	Macedonian	LMR-SMS	(Jovanoski et al., 2015)	mkd	3C	9.71k	1.14k	HF-t
14	Greek	LMR-SMS	(Beleveslis et al., 2019)	gre	3C	1.64k	1.64k	All
15	Danish	LMR-SMS	(Pauli et al., 2021)	dan	3C	3.46k	1.05k	HF-t
16	Norwegian	LMR-SMS	(Oye, 2015)	nor	3C	1,847	1,847	All
17	Swedish	LMR-SMS	(Berggren, 2023)	swe	3C	2,368	2,368	All
18	Czech	LMR-SMS	(Habernal et al., 2013)	cze	3C	9,752	1,950	Test 20%
19	Latvian	LMR-SMS	(Sproǵis and Rikters, 2020)	lav	3C	5.75k	743	HF-t
20	Azerbaijan	LMR-SMS	(Hajili, 2023)	aze	3C	30k	1.5k	HF-t (5%)
21	Malaysian	LMR-SMS	(Juan et al., 2022)	may	3C	7k	1.4k	Test 20%
22	RomanUrdu	LMR-SMS	(Sharf, 2017)	urd	3C	20.2k	2020	Test 10%
23	Amharic	AfriSenti	(Muhammad et al., 2023)	amh	3C	9,483	2,000	HF-t
24	Algerian Arabic	AfriSenti	(Muhammad et al., 2023)	ary	3C	9,762	2,962	HF-t
25	Hausa	AfriSenti	(Muhammad et al., 2023)	hau	3C	22,155	2,652	HF-t (50%)
26	Igbo	AfriSenti	(Muhammad et al., 2023)	ibo	3C	15,718	1,842	HF-t (50%)
27	Kinyarwanda	AfriSenti	(Muhammad et al., 2023)	kin	3C	5,158	1,027	HF-t
28	Moroccan Arabic	AfriSenti	(Muhammad et al., 2023)	arq	3C	3,062	959	HF-t
29	Mozambican Por.	AfriSenti	(Muhammad et al., 2023)	pt-MZ	3C	7,495	1,832	HF-t (50%)
30	Nigerian Pidgin	AfriSenti	(Muhammad et al., 2023)	pcm	3C	10,559	2,078	HF-t (50%)
31	Oromo	AfriSenti	(Muhammad et al., 2023)	orm	3C	2,494	2,097	HF-t
32	Swahili	AfriSenti	(Muhammad et al., 2023)	swa	3C	3,014	749	HF-t
33	Tigrinya	AfriSenti	(Muhammad et al., 2023)	tir	3C	2,400	2,001	HF-t
34	Twi	AfriSenti	(Muhammad et al., 2023)	twi	3C	4,821	950	HF-t
35	Xitsonga	AfriSenti	(Muhammad et al., 2023)	tso	3C	1,264	255	HF-t
36	Yoruba	AfriSenti	(Muhammad et al., 2023)	yor	3C	15,130	2,258	HF-t (50%)

#### 3.1.3 AfriSenti-SemEval

The AfriSenti-SemEval (Muhammad et al., 2023) dataset (SemEval-2023 Task 12: Sentiment Analysis for African Languages) provides annotated tweets for 14 African languages. It is a shared task focused on sentiment analysis, consisting of over 110,000 annotated tweets, which represents one of the largest datasets for sentiment analysis for underrepresented African languages (sourced from Hugging Face^2^).

Table 1 presents a summary of the datasets' key attributes used in this study. The abbreviations used in the columns of the table are as follows:

- ISO Code: ISO 639 language codes (three-letter lowercase abbreviation).
- Cls.T.: Classification Type.
- 3C: Three-class Classification (positive, neutral, or negative).
- HF-t: Hugging Face test set.
- HF-t (50%): 50% random sample of the Hugging Face test set.

### 3.2 Language resource classification framework

#### 3.2.1 Resource classification criteria

The languages in this study were classified into three resource levels: high, medium, and low based on a combination of factors reflecting their digital presence and the availability of linguistic data for NLP development.

**High-resource** languages are characterized by their availability of large amounts of annotated data, significant representation in pretraining datasets, rich tooling ecosystems, and a large digital presence on the web. English, Spanish, French, German, and Arabic are some examples of high-resource languages because of their large speaker bases and established natural language processing ecosystems.

**Medium-resource** languages are characterized by their moderate amount of annotated data and partial representation in pretraining datasets, but lack the same level of tooling and benchmarking as high-resource languages. Swedish, Czech, Greek, and Hindi are some examples of medium-resource languages that have some level of institutional support but still face some coverage gaps.

**Low-resource** languages are characterized by the limited availability of large-scale annotated corpora and their minimal or absent representation in existing LLM pretraining datasets. Estimates based on published language-prevalence statistics indicate that these languages are often substantially underrepresented in large-scale training data. In addition, low-resource languages typically have a scarce digital presence and lack robust linguistic tooling, further constraining the development and evaluation of NLP systems. This category includes languages such as Albanian, Azerbaijani, Bosnian, Croatian, Danish, Latvian, Macedonian, Malaysian, Norwegian, Serbian, Roman Urdu from LMR-SMS, and all 14 languages from AfriSenti. Many of these languages lack standardized orthographies or have multiple competing writing conventions, highlighting the deliberate inclusion of these languages to assess model performance beyond the high-resource bias of existing benchmarks.

The languages used in this study, along with their language families, branches, sub-branches, and estimated number of speakers (in millions) are summarized in Table 2. This information highlights the linguistic diversity of the selected languages and provides context on their classification and global usage. Speaker numbers are primarily based on Ethnologue (SIL International, https://www.ethnologue.com/insights/ethnologue200/). Figures marked with ^*^ are derived from Wikipedia.

**Table 2:** Overview of the languages and their corresponding language families for the datasets.

Language	Lang family	Branch	Sub-branch	No. of speakers (M)
Albanian	Indo-European	Albanian	Albanian	7.5+
Algerian Arabic	Afro-Asiatic	Semitic	Semitic	43.4
Amharic	Afro-Asiatic	Semitic	Semitic	78
Arabic	Afro-Asiatic	Semitic	Semitic	334.9
Azerbaijani	Turkic	Oghuz	Oghuz	23.6
Bosnian	Indo-European	Slavic	South Slavic	3.1*
Croatian	Indo-European	Slavic	South Slavic	6
Czech	Indo-European	Slavic	West Slavic	12.2
Danish	Indo-European	Germanic	North Germanic	5.8
English	Indo-European	Germanic	West Germanic	1,500
French	Indo-European	Romance	Romance	333.5
German	Indo-European	Germanic	West Germanic	133.4
Greek	Indo-European	Hellenic	Hellenic	13.2
Hausa	Afro-Asiatic	Chadic	Chadic	94.5
Hindi	Indo-European	Indo-Aryan	Indo-Aryan	611.2
Igbo	Niger-Congo	Volta-Niger	Volta-Niger	34.4
Italian	Indo-European	Romance	Romance	65.6
Kinyarwanda	Niger-Congo	Bantu	Bantu	15.3
Latvian	Indo-European	Baltic	Baltic	1.75*
Macedonian	Indo-European	Slavic	South Slavic	1.6*
Malaysian	Austronesian	Malayo	Malayo	29.5
Moroccan Arabic	Afro-Asiatic	Semitic	Semitic	40.4
Mozamb. Portuguese	Indo-European	Romance	Romance	13*
Nigerian Pidgin	English Creole	Eng. Creole	Eng. Creole	120.7
Norwegian	Indo-European	Germanic	North Germanic	5.5
Oromo	Afro-Asiatic	Cushitic	Cushitic	48.4
Portuguese	Indo-European	Romance	Romance	269.4
Roman Urdu	Indo-European	Indo-Aryan	Indo-Aryan	70+*
Serbian	Indo-European	Slavic	South Slavic	8.7
Spanish	Indo-European	Romance	Romance	561.3
Swahili	Niger-Congo	Bantu	Bantu	95.2
Swedish	Indo-European	Germanic	North Germanic	11.5
Tigrinya	Afro-Asiatic	Semitic	Semitic	11.2
Twi	Niger-Congo	Kwa	Kwa	10+
Xitsonga	Niger-Congo	Bantu	Bantu	10
Yoruba	Niger-Congo	Volta-Niger	Volta-Niger	50+

#### 3.2.2 Language grouping

**Figure 2:**
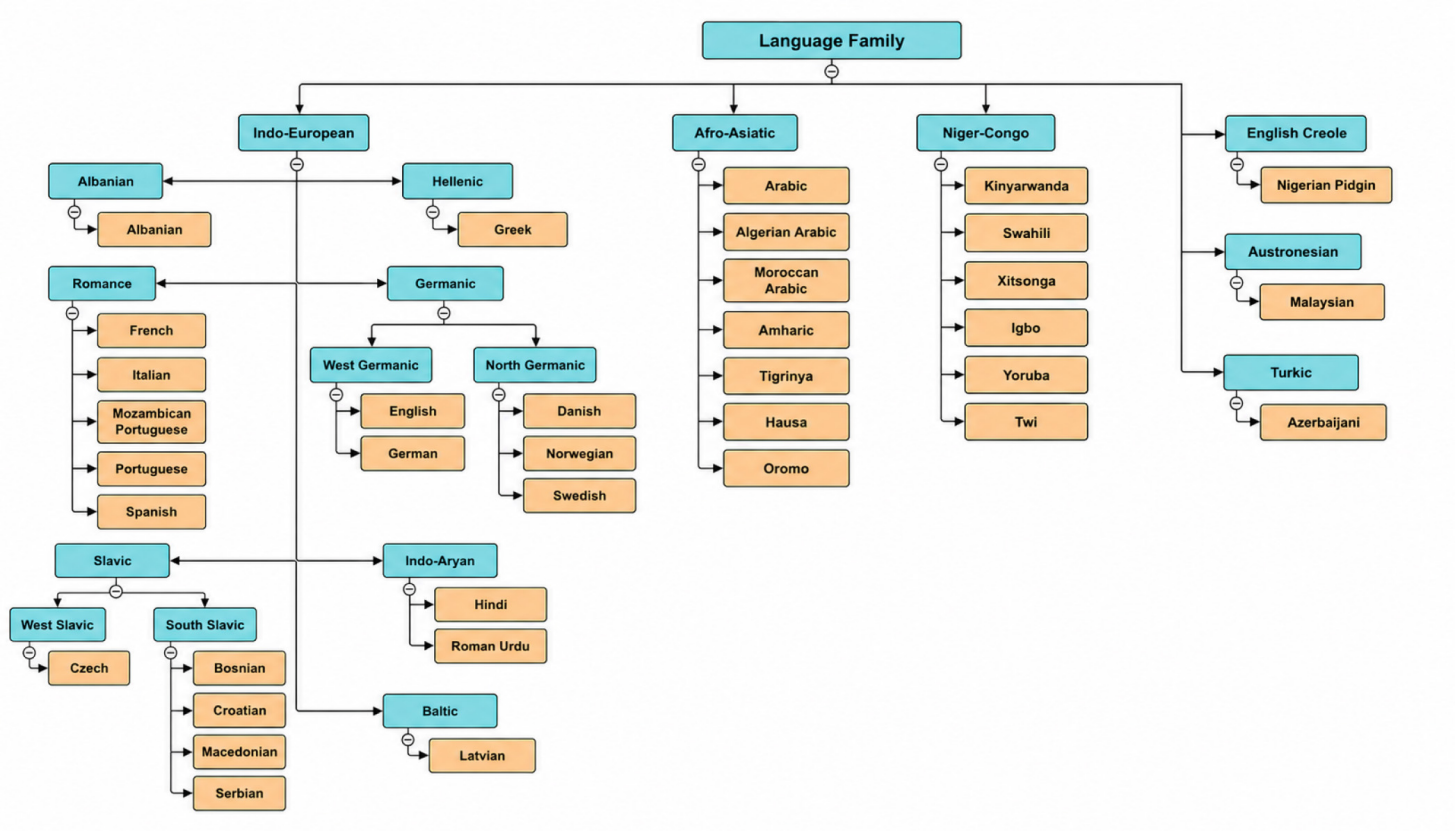
The language family hierarchical tree chart for the languages used in this study.

We applied genealogical classification to group languages based on their roots, as illustrated in Figure 2. The languages are organized into major language families (e.g., Indo-European, Afro-Asiatic, Niger-Congo, Turkic, Uralic, Austronesian, and Creole), represented in light blue/cyan. Large families, such as Indo-European, are further divided into linguistic subgroups, including Romance, Germanic, Indo-Aryan, Slavic, Albanian, Hellenic, and Baltic. Individual languages used in the study appear at the lowest level of the hierarchy, shown in light orange/peach. This structure provides an overview of the linguistic diversity represented in the dataset and clarifies the rationale for grouping languages during analysis.

### 3.3 Model selection

This research employs five state-of-the-art (SOTA) LLMs, including GPT-4o, Gemini 2.0 Flash, DeepSeek-V3, Mistral Large, and Claude 3.7 Sonnet, which cover diverse training data on a large corpus of real-world data, making them forefront in the market at current era. Their selection was guided by several key considerations, including their demonstrated capability in multilingual tasks, the availability of public APIs, and their widespread adoption and practical relevance in real-world applications. The following model name will be utilized throughout the study: GPT-4o, Gemini, DeepSeek, Mistral, and Claude. The specific model versions, release dates, knowledge cutoff dates, and associated API costs are summarized in Table 3.

**Table 3:** Model versions, release dates, knowledge cutoff dates, and API pricing for all evaluated models.

Model	Version	Release date	Knowledge cutoff	Input ($/M tok.)	Output ($/M tok.)
GPT-4o	gpt-4o-2024-08-06	May 2024	Oct 2023	$2.50	$10.00
Gemini	gemini-2.0-flash	Feb 2025	Aug 2024	$0.10	$0.40
DeepSeek	deepseek-chat (V3)	Dec 2024	Mar 2024	$0.27	$1.10
Mistral	mistral-large-2411	Nov 2024	Oct 2023	$2.00	$6.00
Claude	claude-3-7-sonnet-20250219	Feb 2025	Oct 2024	$3.00	$15.00

Costs are denominated in USD per million tokens.

#### 3.3.1 GPT

This research utilized GPT-4o, developed by OpenAI^3^ and released in May 2024 with a knowledge cutoff of October 2023. GPT-4o introduced native multimodal capabilities along its strong multilingual text understanding. We accessed GPT-4o via the API using the gpt-4o-2024-08-06 model snapshot.

#### 3.3.2 Gemini

The study utilized the Gemini 2.0-Flash model, developed by Google DeepMind^4^ and released in February 2025 with a knowledge cutoff of August 2024; it is positioned as a high-efficiency model optimized for speed and cost-effectiveness while retaining strong reasoning and multilingual performance. We accessed this model via the API using the gemini-2.0-flash identifier.

#### 3.3.3 DeepSeek-V3

DeepSeek-v3 is developed by DeepSeek AI^5^, a Chinese AI research laboratory, and released in December 2024 with a knowledge cutoff of March 2024. DeepSeek-V3 is a Mixture-of-Experts (MoE) model notable for its competitive performance relative to its inference cost. We accessed this model via the API using the deepseek-chat endpoint, which corresponds to the DeepSeek-V3 model.

#### 3.3.4 Mistral

Mistral Large (version mistral-large-2411) is developed by Mistral AI^6^. It was released in November 2024 with a knowledge cutoff of October 2023. It represents Mistral AI's flagship dense model, with an emphasis on instruction-following and multilingual text tasks. We accessed this model via the API using the mistral-large-2411 snapshot.

#### 3.3.5 Claude

Claude 3.7 Sonnet, developed by Anthropic^7^, is used in this study. This version was released in February 2025 with a knowledge cutoff of October 2024. Claude 3.7 Sonnet is part of the Claude 3 model family and is notable for its extended thinking capabilities and strong performance on reasoning-intensive tasks. We accessed this model via the API using the claude-3-7-sonnet-20250219 snapshot.

### 3.4 Prompt engineering

All five LLMs were evaluated under both zero-shot and few-shot prompting conditions, with the sentiment classification task framed as a three-class problem: positive, neutral, or negative. Prompting was conducted exclusively via the respective model APIs, with no task-specific fine-tuning applied to any model.

#### 3.4.1 Prompt design

Each prompt consisted of two components: a *system message* and a *user message*. The system message established the model's role as a sentiment classification assistant and specified the output format, constraining responses to a single label from the three permitted classes. The user message provided the input text to be classified. This separation of instructions and input follows established best practices for instruction-tuned models (Sahoo et al., 2024) and promotes consistent outputs across models.

To ensure comparability across models and languages, a single unified prompt template was used for all experiments, with only the input text varying between requests. No model-specific prompt tuning was performed; the same template was applied uniformly across all five LLMs.

#### 3.4.2 Zero-shot prompting

**Figure 3:**
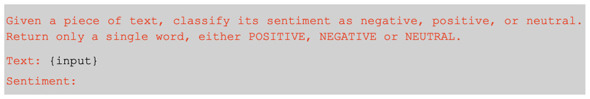
Zero-shot prompt template used for sentiment classification across all five LLMs.

In the zero-shot setting, the model receives only the task instruction and the input text, without any labeled examples. This condition tests the model's intrinsic capacity to perform sentiment classification based solely on its pretraining knowledge and instruction-following ability. The zero-shot prompt template used in this study is illustrated in Figure 3.

#### 3.4.3 Few-shot prompting

**Figure 4:**
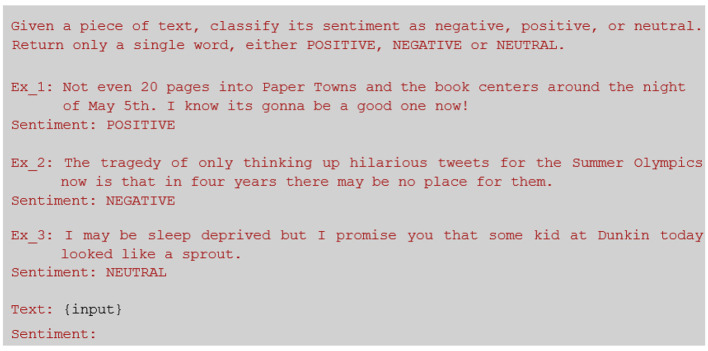
Few-shot prompt template used for sentiment classification across all five LLMs.

In the few-shot setting, the prompt is augmented with a small number of labeled examples prior to the target input. These in-context examples serve to demonstrate the expected input-output format and provide implicit guidance on label usage, without requiring any gradient-based updates to the model. The few-shot prompt template follows the same structural design as the zero-shot template, with the addition of example pairs, as illustrated in Figure 4.

The examples used in few-shot prompts were selected to represent each sentiment class and were kept consistent across all datasets and languages to avoid introducing dataset-specific biases. This design choice ensures that observed differences between zero-shot and few-shot performance can be attributed to the presence of in-context examples rather than example selection artifacts.

### 3.5 Evaluation metrics

To measure the performance of the models employed in this study, the F1 score is used as the primary evaluation metric. The F1 score is a widely adopted performance metric for evaluating classification models' performance, particularly in scenarios where class distributions are imbalanced. Unlike accuracy, the F1 score provides a balanced assessment by considering both false positives and false negatives. It is defined as the harmonic mean of precision and recall, ensuring that a high value is achieved only when both metrics are high.

- **Precision**: The ratio of correctly predicted positive instances to the total number of instances predicted as positive.
- **Recall**: The ratio of correctly predicted positive instances to the total number of actual positive instances.

The F1 score is computed as:


$$\begin{array}{c} F1score=2*\frac{\text{Precision}*\text{Recall}}{\text{Precision}+\text{Recall}} \end{array}$$

Since this study focuses on three-class sentiment classification (positive, negative, and neutral), the F1-score is computed separately for each class by considering one class as the positive class and the remaining classes as negative (one-vs.-rest setting). The overall performance is then obtained by averaging the F1 scores across all classes. This approach provides a more reliable assessment of model performance for multi-class sentiment analysis tasks.

### 3.6 Experimental setup

For each dataset, evaluation was conducted on the available test split as hosted on Hugging Face. In cases where the test set exceeded 3,000 instances, a stratified 50% subsample was applied to balance computational cost against the evaluation coverage while preserving the original class distribution. The exact number of instances used per dataset and language is reported in Table 1.

All five LLMs were queried exclusively via their respective APIs using text-based prompts, as described in Section 3.4. No fine-tuning, adapter layers, or parameter updates of any kind were applied to any model, only a pre-trained baseline model was used. To ensure reproducibility and comparability across models, all generation parameters were held at their API defaults throughout all experiments. In particular, the temperature (*T*), top-*K*, and top-*P* sampling parameters were left at their default values, meaning no manual tuning of decoding behavior was performed. This approach ensures that observed differences in model performance reflect intrinsic model capabilities rather than artifacts of hyperparameter optimization.

To further ensure reliability and traceability, all experiments were conducted using fixed, versioned API snapshots of each model (e.g., gpt-4o-2024-08-06, gemini-2.0-flash, deepseek-chat, mistral-large-2411, and claude-3-7-sonnet-20250219), preventing result variability that could arise from model updates or redeployments over time. Furthermore, a single unified prompt template was applied uniformly across all five models and all 36 languages, ensuring that observed performance differences reflect intrinsic model capabilities rather than prompt-specific artifacts. These design choices are consistent with established practices in LLM evaluation literature (Chang et al., 2024; Zhang et al., 2023) and are explicitly documented to support full reproducibility.

Each input was submitted as an independent, stateless API call with no conversation history carried across instances. All experiments were conducted programmatically, with model responses parsed and mapped to one of the three sentiment classes, positive, neutral, or negative using an exact string match against the expected label set. Responses that could not be mapped to a valid label were flagged and handled as described in Section 3.5.

## 4 Results

To better understand the multilingual capabilities of the evaluated LLMs, we analyzed results according to the linguistic families illustrated in Figure 2. Grouping languages by genealogical relationships reveals clear trends in model behavior across related languages and provides insights into how linguistic similarity influences sentiment analysis performance.

### 4.1 Indo-European languages—results

The Indo-European family is the largest and most diverse group in the evaluation, comprising the Albanian, Hellenic, Romance, Germanic, Slavic, Indo-Aryan, and Baltic branches. The performance of the five pretrained LLMs assessed on this group of languages is shown in Table 4. Results are reported for both zero-shot and few-shot prompting settings, and the state-of-the-art benchmark is included for reference. As shown in Table 4, the models achieved their strongest and most consistent performance within this family, indicating that Indo-European languages are comparatively well represented in LLM pretraining corpora.

**Table 4:** F1 scores (%) of LLMs for sentiment classification across the Indo-European language family.

Dataset	Lang	Prompt	GPT-4o	Gemini	DeepSeek	Mistral	Claude	SOTA
Albanian	alb	Zero-Shot	69.8	53.4	54.0	50.3	64.8	*72.1*
		Few-Shot	**70.2**	70.0	63.6	53.8	65.4	
Greek	gre	Zero-Shot	82.9	84.7	83.0	81.2	82.1	*80.6*
		Few-Shot	86.1	86.0	85.1	***89.3***	88.3	
French	fra	Zero-Shot	55.8	64.9	58.2	51.7	52.6	*68.2*
		Few-Shot	65.1	63.2	48.7	**66.3**	63.0	
Italian	ita	Zero-Shot	79.5	75.9	71.1	63.9	79.4	*70.9*
		Few-Shot	79.9	80.2	77.3	***80.5***	79.9	
Portuguese	por	Zero-Shot	79.7	67.2	73.8	68.5	74.1	*76.0*
		Few-Shot	76.8	***80.5***	74.2	68.1	73.5	
M. Portuguese	pt-MZ	Zero-Shot	67.5	43.0	29.4	58.6	52.8	*75.0*
		Few-Shot	**72.5**	52.2	65.5	59.5	50.1	
Spanish	spa	Zero-Shot	73.7	61.1	70.3	63.8	68.0	*68.5*
		Few-Shot	***71.9***	70.5	66.8	64.2	65.2	
English	eng	Zero-Shot	70.1	65.1	71.9	62.5	73.3	*66.9*
		Few-Shot	70.8	66.4	69.7	68.4	***72.4***	
German	deu	Zero-Shot	71.0	61.4	70.8	60.9	75.6	*76.1*
		Few-Shot	74.8	68.6	75.1	76.0	***77.2***	
Danish	dan	Zero-Shot	68.7	66.3	69.0	58.7	72.1	*63.7*
		Few-Shot	70.5	68.2	71.1	70.8	***72.7***	
Norwegian	nor	Zero-Shot	76.8	61.6	57.1	64.8	72.5	*71.0*
		Few-Shot	***74.4***	70.1	71.4	69.8	69.9	
Swedish	swe	Zero-Shot	78.7	72.9	69.1	66.4	76.7	*47.0*
		Few-Shot	***79.6***	76.1	76.6	78.5	75.5	
Bosnian	bos	Zero-Shot	58.3	54.6	55.6	43.8	59.2	*60.6*
		Few-Shot	61.7	61.2	62.2	61.3	***64.4***	
Croatian	hrv	Zero-Shot	50.5	55.1	56.3	37.6	53.5	*60.6*
		Few-Shot	56.6	53.3	59.4	***63.5***	61.9	
Serbian	srb	Zero-Shot	58.1	50.1	47.6	45.9	57.1	*60.6*
		Few-Shot	**59.1**	56.3	56.5	51.8	54.5	
Macedonian	mkd	Zero-Shot	63.7	66.0	63.1	43.2	65.8	63.0*
		Few-Shot	65.3	68.6	***71.5***	68.8	70.6	
Czech	cze	Zero-Shot	80.0	67.3	55.3	63.9	69.9	*69.0*
		Few-Shot	***76.4***	75.7	66.4	63.9	67.5	
Hindi	hin	Zero-Shot	66.9	65.6	62.8	54.0	65.8	*40.3*
		Few-Shot	67.4	***68.4***	65.1	64.3	65.4	
Roman Urdu	urd	Zero-Shot	64.9	61.4	54.4	48.2	59.6	*68-80*
		Few-Shot	**65.7**	63.3	61.8	58.5	62.7	
Latvian	lav	Zero-Shot	71.1	68.0	64.2	47.9	67.7	*61.2*
		Few-Shot	72.8	***73.2***	69.9	67.7	70.7	

Underline, best zero-shot; Italic, SOTA; Bold, best few-shot; Bold + Italic, few-shot surpassing SOTA.

#### 4.1.1 Romance languages

The Romance subgroup includes French, Italian, Portuguese, Mozambican Portuguese, and Spanish. Among these languages, Italian, Portuguese, and Spanish achieved high performance by the evaluated models. For Italian, the highest performance is obtained by Mistral in the few-shot setting, achieving an F1 score of 80.5%, followed closely by Gemini with an F1 score of 80.2% and GPT-4o and Claude with 79.9%. These results substantially exceed the reported SOTA F1 score of 70.9%. Similarly, Spanish demonstrated high performance, with GPT-4o achieving the highest F1 score of 73.7% in the zero-shot setting and 71.9% in the few-shot setting, both surpassing the SOTA F1 score of 68.5%.

Portuguese also demonstrated strong performance across models. Gemini achieved the highest F1 score of 80.5% in the few-shot setting, outperforming the reported SOTA F1 score of 76.0%. GPT-4o also achieved competitive performance with a zero-shot F1 score of 79.7%. In contrast, Mozambican Portuguese proved more challenging. The highest F1 score for this dataset was achieved by GPT-4o in the few-shot setting with 72.5%, which remained below the SOTA F1 score of 75.0%, although it was still the highest result among the evaluated LLMs. French showed comparatively lower F1 scores across all models. Mistral achieved the highest F1 score of 66.3% in the few-shot setting, followed closely by GPT-4o with an F1 score of 65.1%. However, all evaluated models remained below the reported SOTA F1 score of 68.2%. These findings indicate that linguistic similarity within the Romance subgroup does not necessarily guarantee uniformly high sentiment classification performance.

The overall results suggest that all evaluated models handle Romance languages effectively. This could happen because of the extensive representation of these languages in multilingual training data. Additionally, the relatively consistent improvements in F1 scores across most Romance languages indicate strong cross-lingual transfer capabilities within the subgroup.

#### 4.1.2 Germanic languages

The Germanic branch consists of West Germanic languages (English and German) and North Germanic languages (Danish, Norwegian, and Swedish). This subgroup achieved some of the strongest overall F1 scores in the study.

German achieved particularly strong performance, with Claude obtaining the highest F1 score of 77.2% in the few-shot setting, slightly surpassing the reported SOTA F1 score of 76.1%. GPT-4o also achieved competitive results with a few-shot F1 score of 74.8%. Similarly, Swedish demonstrated remarkable improvements, where GPT-4o achieved the highest F1 score of 79.6% in the few-shot setting compared with a substantially lower SOTA F1 score of 47.0%. Danish and Norwegian also demonstrated significant improvement over existing benchmarks. For Danish, Claude achieved the highest F1 score of 72.7% in the few-shot setting, clearly outperforming the SOTA F1 score of 63.7%. For Norwegian, GPT-4o achieved the best F1 score of 74.4% in the few-shot setting, surpassing the SOTA F1 score of 71.0%.

English showed relatively balanced F1 scores across all models. Claude achieved the highest F1 score of 73.3% in the zero-shot setting, outperforming the SOTA F1 score of 66.9%. However, unlike the Scandinavian languages, the improvements over the SOTA baseline for English were comparatively moderate. This could have happened because English sentiment analysis benchmarks are already highly optimized and mature.

The consistently high F1 scores across Germanic languages indicate that LLMs are highly robust for languages with rich resources and extensive representation during pretraining. Furthermore, the relatively small performance gap between zero-shot and few-shot settings suggests that these languages require limited task-specific adaptation.

### 4.2 African language families–results

The African language datasets include languages from the Afro-Asiatic and Niger-Congo families, together with Nigerian Pidgin, which is treated separately as an English Creole. The results summarized in Table 5 show a more varied performance pattern compared to that observed for many Indo-European languages. While some languages outperformed the reported SOTA benchmark, others remained challenging for all evaluated LLMs.

**Table 5:** F1 scores (%) of LLMs for sentiment classification across African language families.

Dataset	Lang	Prompt	GPT-4o	Gemini	DeepSeek	Mistral	Claude	SOTA
Amharic	amh	Zero-Shot	74.7	76.1	75.2	56.7	75.5	*78.4*
		Few-Shot	76.4	78.7	77.4	71.1	***79.3***	
Arabic	ara	Zero-Shot	71.0	72.7	67.9	52.1	67.2	*67.7*
		Few-Shot	70.2	73.4	70.4	71.4	***75.1***	
Alger. Arabic	ary	Zero-Shot	58.6	54.8	46.3	30.9	61.8	*64.8*
		Few-Shot	65.8	***66.5***	55.6	48.1	66.0	
Morocc. Arabic	arq	Zero-Shot	65.6	74.1	64.3	44.0	63.7	*74.2*
		Few-Shot	69.2	**71.4**	66.5	67.0	70.4	
Tigrinya	tir	Zero-Shot	61.4	64.5	57.9	35.3	67.5	–
		Few-Shot	63.2	**72.5**	62.6	50.0	71.9	
Hausa	hau	Zero-Shot	63.7	49.5	50.2	33.2	52.5	*82.6*
		Few-Shot	72.3	**72.6**	65.0	57.7	67.9	
Oromo	orm	Zero-Shot	44.1	35.3	33.1	36.3	43.3	–
		Few-Shot	**45.8**	41.6	44.4	38.0	38.6	
Kinyarwanda	kin	Zero-Shot	67.7	65.6	50.6	40.5	60.7	*72.6*
		Few-Shot	**70.8**	70.2	59.4	52.7	63.8	
Swahili	swa	Zero-Shot	62.0	48.3	42.8	43.5	59.2	65.7
		Few-Shot	**61.6**	58.3	53.3	48.4	53.2	
Xitsonga	tso	Zero-Shot	33.8	40.3	45.0	23.1	35.1	*60.7*
		Few-Shot	46.6	**55.5**	42.9	50.5	54.4	
Twi	twi	Zero-Shot	46.2	44.3	43.5	26.4	39.4	*68.3*
		Few-Shot	51.9	52.9	38.3	49.8	**60.8**	
Igbo	ibo	Zero-Shot	64.9	50.4	51.8	38.9	51.7	*83.0*
		Few-Shot	**71.4**	63.5	60.9	59.0	63.1	
Yoruba	yor	Zero-Shot	60.4	61.2	53.4	27.9	50.4	*80.2*
		Few-Shot	73.1	74.7	60.9	60.5	**78.6**	
Niger. Pidgin	pcm	Zero-Shot	63.4	67.2	63.5	50.6	67.2	*76.0*
		Few-Shot	69.7	73.9	69.7	69.5	**74.4**	

Underline, best zero-shot; Italic, SOTA; Bold, best few-shot; Bold + Italic, few-shot surpassing SOTA.

#### 4.2.1 Afro-Asiatic languages

The Afro-Asiatic group includes Amharic, Arabic, Algerian Arabic, Moroccan Arabic, Tigrinya, Hausa, and Oromo. Within this family, the Semitic languages generally achieved higher F1 scores than other subgroups of languages.

Amharic yielded one of the best results in this group. Claude achieved the highest F1 score of 79.3% in the few-shot setting, surpassing the reported SOTA F1 score of 78.4%. Gemini also showed similar performance, achieving an F1 score of 78.7% in the few-shot setting. This indicates that LLMs can be highly competitive for Amharic sentiment analysis when task examples are provided.

Arabic also showed similar performance. Claude achieved the best F1 score of 75.1% in the few-shot setting, clearly outperforming the reported SOTA F1 score of 67.7%. Gemini obtained the highest F1 score of 72.7% in zero-shot, suggesting that Arabic benefits from both strong pretrained multilingual representations and an additional few-shot strategy.

For Algerian Arabic, the highest F1 score is achieved by Gemini in the few-shot setting, with an F1 score of 66.5%, followed closely by Claude with 66.0% and GPT-4o with 65.8%. These results are slightly higher than the reported SOTA F1 score of 64.8%. Moroccan Arabic demonstrated a different pattern. Gemini achieved the strongest zero-shot F1 score of 74.1%, which is very close to the SOTA F1 score of 74.2%, but the best few-shot result, also from Gemini, dropped to 71.4%. This suggests that few-shot prompting did not improve performance for Moroccan Arabic and may have introduced some instability.

Tigrinya demonstrated a similar pattern. Gemini achieved the highest F1 score of 72.5% in the few-shot setting, followed by Claude with 71.9%. Since no SOTA benchmark results is reported for Tigrinya, these results provide a useful reference point for future comparison.

Hausa and Oromo are more challenging. For Hausa, Gemini achieved the best F1 score of 72.6% in the few-shot setting, closely followed by GPT-4o with 72.3%. However, these results are lower than the reported SOTA F1 score of 82.6%. Oromo showed the lowest results in the Afro-Asiatic group. GPT-4o outperformed other models, with an F1 score of 45.8% in the few-shot setting. This low performance could be due to lower resource availability and limited representation in multilingual pretraining data.

#### 4.2.2 Niger-Congo languages

The Niger-Congo family includes Kinyarwanda, Swahili, Xitsonga, Twi, Igbo, and Yoruba. Compared with the Afro-Asiatic languages, the Niger-Congo group showed greater variability and generally lower F1 scores relative to the reported SOTA baselines.

Kinyarwanda achieved its highest F1 score with GPT-4o in the few-shot setting, reaching 70.8%. Gemini followed closely with an F1 score of 70.2%. Although these scores are competitive, they are lower than the reported SOTA F1 score of 72.6%. Swahili was also challenging: GPT-4o achieved the highest F1 score of 62.0% in the zero-shot setting, while its few-shot F1 score slightly decreased to 61.6%. Both results are lower than the SOTA F1 score of 65.7%, suggesting that additional examples did not necessarily improve the classification of the Swahili sentiment.

For Xitsonga, Gemini achieved the highest F1 score of 55.5% in the few-shot setting, followed by Claude with 54.4%. However, these scores remained below the SOTA F1 score of 60.7%. Twi showed a similar pattern, with Claude achieving the best few-shot F1 score of 60.8%, still lower than the SOTA F1 score of 68.3%.

Igbo and Yoruba showed greater few-shot improvements, particularly with GPT-4o and Claude. For Igbo, GPT-4o achieved the highest F1 score of 71.4% in the few-shot setting, outperforming the other LLMs but remaining below the SOTA F1 score of 83.0%. Yoruba achieved its best result with Claude. It achieved an F1 score of 78.6% in the few-shot setting, which is still slightly lower than the reported SOTA F1 score of 80.2%.

Nigerian Pidgin is difficult to classify because, although its lexifier is English, many of its structural, pragmatic, and sociolinguistic features are shaped by West African languages. As an English-based creole, it does not fit well with either the West Germanic languages or the African language groups analyzed above. It is therefore analyzed separately. The Nigerian Pidgin showed competitive but still lower results compared to the SOTA benchmark. Claude achieved the highest F1 score of 74.4% in the few-shot setting, followed by Gemini with 73.9%. However, these results are lower than the reported SOTA F1 score of 76.0%.

### 4.3 Austronesian and Turkic language families—results

This section presents a sentiment analysis on Malaysian from the Austronesian language family and Azerbaijani from the Turkic language family. The overall F1 scores for these languages are significantly lower across all evaluated LLMs, as shown in Table 6. However, various patterns are identified regarding model behavior and prompting strategies.

**Table 6:** F1 scores (%) of LLMs for sentiment analysis across two other language families.

Dataset	Lang	Prompt	GPT-4o	Gemini	DeepSeek	Mistral	Claude	SOTA
Azerbaijani	aze	Zero-Shot	41.1	37.5	37.1	32.7	38.3	–
		Few-Shot	42.0	39.0	37.3	37.6	**43.3**	
Malaysian	may	Zero-Shot	41.4	47.4	49.7	39.9	49.1	–
		Few-Shot	41.7	46.8	48.8	44.4	**50.5**	

Underline, best zero-shot; Bold, best few-shot.

Azerbaijani represents the Turkic language family in this evaluation. It proves challenging for all evaluated models, with F1 scores remaining relatively low in both zero-shot and few-shot settings. Among the evaluated models, Claude achieved the highest F1 score of 43.3% in the few-shot setting, followed closely by GPT-4o with 42.0%.

Malaysian, representing the Austronesian language family, achieved slightly higher F1 scores than Azerbaijani, but lower compared with other larger language families analyzed above in this section. Claude achieved the highest F1 score of 50.5% in the few-shot setting, followed closely by DeepSeek with 48.8% and Gemini with 46.8%. Interestingly, DeepSeek achieved the highest zero-shot F1 score of 49.7%, outperforming all other models in this setting.

Unlike many evaluated languages, Malaysian showed relatively small differences between zero-shot and few-shot settings. For instance, GPT-4o, achieved nearly identical results in both settings, with F1 scores of 41.4% and 41.7%, respectively.

### 4.4 Error analysis by language family

To identify systematic misclassification patterns beyond aggregate macro F1 scores, we conducted a per-class error analysis across all 36 languages, computing class-level F1 scores for the positive, negative, and neutral sentiment classes separately and aggregating results at the language-family level. The findings, summarized in Table 7, show consistent and linguistically interpretable patterns across all five models.

**Table 7:** Average per-class F1 scores (%) by language family, aggregated across all five models.

Language family	Languages	Macro F1	Positive	Negative	Neutral	Neg–Neu gap
Indo-European	20	**67.3**	**67.8**	73.9	**60.3**	13.6
Afro-Asiatic	7	61.1	62.9	71.2	49.3	21.9
Niger-Congo	6	55.1	57.0	56.9	51.4	5.5
English Creole	1	61.7	63.1	**82.6**	39.4	**43.2**
Turkic	1	39.7	27.7	51.9	39.7	12.2
Austronesian	1	46.1	38.0	58.4	41.9	16.5
**Overall**	**36**	62.7	62.8	69.2	54.5	14.7

Bold values indicate the highest score achieved in each column.

Across all six language families, the neutral class consistently yields the lowest F1 scores, making it the primary driver of macro F1 degradation for all five models. As shown in Table 7, the gap between negative and neutral class is substantial in every family, with the largest disparities observed in English Creole (43.2 percentage points), Afro-Asiatic (21.9 percentage points), and Austronesian (16.5 percentage points). This systematic bias reflects a well-known challenge in three-class sentiment classification: neutral expressions are contextually ambiguous, often lack distinctive lexical markers, and require pragmatic inference that current LLMs struggle to perform reliably in low-resource language settings (Zhang et al., 2023). At the individual language level, Twi (27.0%), Xitsonga (33.0%), and Oromo (36.2%) exhibit the most severe neutral-class underperformance across all models. At the same time, Greek stands out as a remarkable outlier with an average F1 score of 90.9% on neutral, the highest across all 36 languages.

In contrast, the negative class consistently achieves the highest F1 scores across all six language families, with English Creole reaching an average of 82.6%, Indo-European 73.9%, and Afro-Asiatic 71.2%. This cross-family advantage for negative sentiment detection is likely attributable to the prevalence and lexical distinctiveness of negative expressions in social media text, as well as the disproportionate representation of negative content in pretraining corpora (Wankhade et al., 2022). In Nigerian Pidgin specifically, the strong performance on the negative class (82.6%) is amplified by frequent code-mixing with English, which provides recognizable negative sentiment anchors even for models with limited Nigerian Pidgin pretraining coverage.

Among all language families, Indo-European languages demonstrate the most consistent per-class F1 scores across all three sentiment classes, with average positive (67.8%), negative (73.9%), and neutral (60.3%) F1 values substantially higher than all other families. This advantage reflects the richer digital corpora, greater pretraining representation, and more standardized orthographies of Indo-European languages. Notably, even within this family, low-resource South Slavic languages such as Croatian (Neutral: 43.6%) and Macedonian (neutral: 38.8%) show markedly weaker neutral-class performance than high-resource members such as Italian (neutral: 74.7%) and German (neutral: 67.2%), underscoring that resource level is a stronger predictor of per-class performance than language family membership alone.

A different pattern emerges in the Niger-Congo family, where models show uniformly weak performance across all sentiment classes rather than a strong bias toward the negative class. The F1 scores of 57.0%, 56.9%, and 51.4% for positive, negative and neutral classes, respectively, fall within a narrow 5.5 percentage point range. This pattern suggests that models fail uniformly across all sentiment classes in Niger-Congo languages rather than being systematically biased toward negative detection. This may reflect the combination of tonal language properties, morphological complexity (particularly in Bantu languages such as Kinyarwanda and Swahili), and the near-complete absence of these languages from standard LLM pretraining corpora (Lupascu et al., 2025; García-Díaz et al., 2023).

The Turkic family (Azerbaijani) and the Austronesian family (Malaysian) show another distinctive trend. They perform very low on the positive class with F1 scores of 27.7% and 38.0%, respectively, compared to their negative-class performance (51.9% and 58.4%). This extreme asymmetry suggests that LLMs have internalized virtually no reliable lexical or structural cues for positive sentiment expression in these languages, which are characterized by agglutinative morphology (Azerbaijani) and complex discourse-level positivity markers (Malaysian) that differ fundamentally from the Indo-European patterns dominant in pretraining data (Lupascu et al., 2025).

#### 4.4.1 Model-level error analysis

Across all 36 languages, as shown in Table 8, GPT-4o achieves the highest average F1 score of 59.7% on the neutral class, demonstrating the strongest ability to distinguish ambiguous sentiment contexts. Claude leads the positive class with an F1 score of 67.5%, suggesting stronger sensitivity to positive sentiment markers across diverse languages. Mistral shows the weakest performance on neutral class (F1 score of 48.8%), consistent with its overall weaker zero-shot performance noted in Section 4.1. DeepSeek achieves a competitive performance on neutral with an F1 score of 55.6% despite its substantially lower API cost, reinforcing its cost-efficiency advantage identified in Section 5.2. Notably, both GPT-4o and Gemini produce zero invalid (unparseable) responses across all 36 languages, while Claude, DeepSeek, and Mistral show marginal invalid response rates of approximately 0.1%–0.2%, indicating robust instruction-following across all evaluated models.

**Table 8:** Average F1 scores (%) achieved by models across three classes of sentiment.

Model	Macro F1	Positive	Negative	Neutral
GPT-4o	**64.7**	63.0	**71.4**	**59.7**
Claude	64.1	**67.5**	70.8	54.0
Gemini	63.7	63.3	71.2	56.6
DeepSeek	61.2	60.3	67.8	55.6
Mistral	58.8	60.3	67.3	48.8

Bold values indicate the highest score achieved in each column.

## 5 Discussion

### 5.1 Effect of few-shot prompting on model performance

The results show that few-shot prompting generally leads to substantial performance improvements compared to zero-shot prompting and often reaches or surpasses the state-of-the-art results achieved by models explicitly trained on those datasets. These improvements are particularly notable in low-resource languages. Models that perform comparatively weaker in zero-shot settings benefit most from few-shot prompting. Mistral, for instance, transitions from underperforming in zero-shot mode to becoming competitive once a few-shot examples are provided.

However, in a few high-resource languages, GPT-4o does not show notable performance improvements. In some languages, such as Arabic, Portuguese, and Spanish, the performance even drops slightly when moving from zero-shot to few-shot prompting. Similar effects are observed for Claude in certain high-resource languages. This may have happened because these models already possess strong task understanding and follow natural-language instructions effectively without additional examples. Providing few-shot samples in such cases may introduce noise or distract the model, leading it to overfit to the specific patterns of the examples or rely on superficial linguistic cues rather than true sentiment. For instance, if the few-shot samples contain short, simple tweets while the evaluation input is longer or code-mixed, the model may incorrectly focus on features like message length or emoji usage. As a result, zero-shot prompting can sometimes offer more stable and generalizable performance for sentiment classification, especially in some high-resource languages and certain challenging low-resource scenarios.

It is interesting to note that few-shot prompting helps significantly to narrow the performance gap between high-resource and low-resource languages, although some underrepresented languages, including Azerbaijani, Malaysian, Oromo, Twi, and Xitsonga, remain challenging. Nonetheless, the results demonstrate that few-shot prompting is a cost-effective and impactful method for boosting LLM performance, particularly in settings where training data or computational resources are limited.

### 5.2 Linguistic reasons behind performance gaps

The performance disparities observed across language families reflect a combination of structural linguistic properties and pretraining data availability. Within the Indo-European family, within-group variation is driven primarily by resource level rather than linguistic complexity. Low-resource South Slavic languages such as Serbian (55.6%) and Croatian (58.9%) lag considerably behind high-resource members such as Greek (87.0%) and Italian (79.6%), despite broadly similar morphosyntactic structures (Lupascu et al., 2025).

In the Afro-Asiatic family, dialectal Arabic varieties (Algerian and Moroccan Arabic) underperform relative to Modern Standard Arabic due to substantial differences in vocabulary and morphology that LLMs trained predominantly on MSA cannot bridge (Zhang et al., 2023). Oromo's severe underperformance (41.7%) reflects its Cushitic branch isolation and near-complete absence from pretraining corpora (Koto et al., 2024).

Niger-Congo languages exhibit a uniquely uniform cross-class failure with positive, negative, and neutral F1 scores clustered within 5.5 points, attributable to complex noun class systems, agglutinative morphology, and tonal properties in Bantu and Kwa languages that are absent from text-based tokenization (Aliyu et al., 2024; García-Díaz et al., 2023). Nigerian Pidgin (English Creole) achieves strong negative F1 (82.6%) through its English lexifier advantage, but collapses on neutral classification (39.4%) where substrate-influenced pragmatic conventions diverge from English (Sibitenda et al., 2024).

Finally, Azerbaijani and Malaysian represent the most severe cases of structural incompatibility, where agglutinative morphology and near-complete pretraining absence combine to produce catastrophic positive-class F1 scores of 27.7% and 38.0%, respectively, the lowest of any language-class combination in this study (Lupascu et al., 2025; Chang et al., 2024).

### 5.3 Cost analysis

Table 9 shows the cost of the models used per dataset (language). As can be seen from the table, the three models, GPT-4o, Mistral, and Claude, have an approximate cost that is many times higher compared to the other two models, Gemini and DeepSeek. Although Gemini and DeepSeek have a significantly lower cost than the other models, in many cases, they provide almost the same performance, especially Gemini, which, in several datasets (languages), outperforms the other models.

**Table 9:** Cost of models per dataset (in USD).

Dataset	No. of instances	GPT4o	Gemini	DeepSeek	Mistral	Claude
Norwegian	1,841	$1.25	$0.04	$0.05	$1.09	$1.69
Swedish	2,368	$1.48	$0.11	$0.07	$2.44	$2.05
Czech	1,950	$1.37	$0.06	$0.06	$1.22	$1.75
Amh & Kin	3,027	$4.34	$0.17	$0.11	$4.58	$4.30
Slovak	1,100	$0.60	$0.04	$0.03	$0.58	$0.84

### 5.4 Current limitations

We acknowledge a few limitations of this study that could be addressed in future research. The study focuses on a limited number of languages for which publicly available datasets exist and compares the performance of only five prominent pretrained LLMs. This section briefly presents the limitations of our work.

- Although this study spans 36 languages across diverse linguistic and cultural contexts, it is not possible to cover all languages spoken worldwide. Many low-resource languages spoken by millions of people still lack publicly available datasets, which remains a major gap in current LLM research.
- The study relies primarily on Twitter/X datasets for sentiment analysis, with only two datasets sourced from Facebook. Data from other social media platforms, such as YouTube, Instagram, WhatsApp, and others, are not included, as there are no publicly available datasets.
- Dataset sizes, class distributions, and testing samples are not uniform across languages, which may affect the comparability of results.
- This work evaluates only five prominent pretrained LLMs (GPT-4o, Gemini, DeepSeek, Mistral, and Claude) and does not assess other models such as LLaMA or newer LLMs currently available. As a result, other models may perform better on the datasets and languages considered in this study.
- A further limitation concerns potential data contamination. Since all five evaluated LLMs are closed-source models trained on large, partially undisclosed pretraining corpora, it cannot be fully verified whether any of the benchmark datasets used in this study were included during pretraining. This is a known challenge in evaluating frontier LLMs on public benchmarks and may partially inflate performance estimates, particularly for widely used datasets such as TSM and AfriSenti-SemEval. Future work should consider evaluating on held-out or newly collected datasets that postdate the knowledge cutoff of the evaluated models to mitigate this risk.

### 5.5 Practical implications

The findings of this study carry several practical implications for researchers, developers, and policymakers working at the intersection of sentiment analysis and multilingual LLMs. First, the strong performance of few-shot prompting across most languages, including several low-resource ones, suggests that practitioners working in resource-constrained settings can achieve meaningful improvements without the need for expensive fine-tuning or annotated training data, simply by designing effective in-context examples. Second, the cost analysis reveals that Gemini and DeepSeek offer competitive performance at a fraction of the cost of GPT-4o, Mistral, and Claude, making them particularly attractive for large-scale multilingual deployments in low-budget settings such as public health monitoring, educational feedback systems, and governance applications in developing regions. Third, the persistent underperformance on languages such as Oromo, Xitsonga, Azerbaijani, and Twi highlights the urgent need for LLM developers to invest in more diverse and representative pretraining corpora, particularly for African and Turkic languages. Finally, the hierarchical language-family analytical framework introduced in this study provides a reusable evaluation lens that future multilingual NLP benchmarks can adopt to move beyond flat, per-language reporting toward more structured, genealogically informed assessments of model coverage.

## 6 Conclusion and future work

This study provides a comprehensive evaluation of five contemporary LLMs across 36 datasets representing 36 languages for multilingual sentiment analysis. The findings indicate that current LLMs achieve strong and often competitive performance across a wide range of linguistic settings, particularly for high- and medium-resource languages. Few-shot prompting consistently enhances performance across most evaluated languages, with several models matching or surpassing existing state-of-the-art benchmarks. Although Mistral demonstrates comparatively weaker zero-shot performance, it benefits substantially from few-shot prompting and becomes highly competitive under in-context learning settings.

Despite these encouraging results, performance disparities across language groups remain substantial. Models such as GPT-4o, Claude, and Gemini achieve robust and reliable results for high- and medium-resource languages but their performances decrease considerably for many low-resource languages. Azerbaijani, Malaysian, Oromo, Twi, and Xitsonga remain particularly challenging across both prompting settings. These findings underscore the persistent imbalance in multilingual NLP and highlight the need for more inclusive pretraining corpora, better linguistic representation, and evaluation benchmarks that more accurately reflect the world's linguistic diversity. Many low-resource languages remain significantly underrepresented in online content despite being spoken by large populations globally.

This study also demonstrates the value of analyzing multilingual performance through a language-family perspective rather than relying exclusively on per-language comparisons. The hierarchical analysis reveals systematic variations across genealogically related languages, suggesting that linguistic proximity and representation in training data influence cross-lingual generalization capabilities.

Future work will focus on fine-tuning the best-performing and computationally efficient models using supervised datasets to further examine the impact of task-specific adaptation. We also plan to incorporate Explainable AI techniques to better understand model behavior across different linguistic settings, particularly to investigate why models such as Mistral benefit more substantially from few-shot prompting and why low-resource languages continue to present major challenges for all evaluated models. In addition, future studies should extend the evaluation to a broader range of emerging LLMs and multilingual benchmarks to provide a more comprehensive understanding of cross-lingual sentiment analysis performance.

## Data Availability

The raw data supporting the conclusions of this article will be made available by the authors, without undue reservation.

## Footnotes

1 https://huggingface.co/datasets/cardiffnlp/tweet_sentiment_multilingual (Accessed May 16, 2025).

2 https://huggingface.co/datasets/shmuhammad/AfriSenti-twitter-sentiment (Accessed March 13, 2025).

3
https://openai.com/index/hello-gpt-4o/

4
https://ai.google.dev/gemini-api/docs/models#gemini-2.0-flash

5
https://api-docs.deepseek.com/quick_start/pricing/

6
https://docs.mistral.ai/getting-started/changelog/

7
https://www.anthropic.com/news/claude-3-7-sonnet
